# Tacrolimus Concentration after Letermovir Initiation in Hematopoietic Stem Cell Transplantation Recipients Receiving Voriconazole: A Retrospective, Observational Study

**DOI:** 10.7150/ijms.42011

**Published:** 2020-03-15

**Authors:** Shinichi Hikasa, Shota Shimabukuro, Yuko Osugi, Kazuhiro Ikegame, Katsuji Kaida, Keiko Fukunaga, Tomoko Higami, Masami Tada, Kuniyoshi Tanaka, Mina Yanai, Takeshi Kimura

**Affiliations:** 1 Department of Pharmacy, Hyogo College of Medicine College Hospital, Nishinomiya, Hyogo 663-8501, Japan; 2 Division of Hematology, Department of Internal Medicine, Hyogo College of Medicine, Nishinomiya, Hyogo 663-8501, Japan

**Keywords:** Letermovir, tacrolimus, voriconazole, interaction, hematopoietic stem cell transplantation

## Abstract

Letermovir (LMV) is a new antiviral drug used to prevent cytomegalovirus infection in hematopoietic stem cell transplantation (HSCT) recipients. It has been reported to increase tacrolimus (TAC) exposure and decrease voriconazole (VRCZ) exposure in healthy participants. However, VRCZ inhibits the metabolism of TAC. Thus, the effects of LMV on TAC exposure in patients receiving VRCZ are unknown. This retrospective, observational, single-center study was conducted between May 2018 and April 2019. The TAC concentration/dose (C/D) ratio, VRCZ concentration, and VRCZ C/D ratio for 7 days before and for the first and second 7-day periods after the initiation of LMV administration were evaluated. Fourteen HSCT recipients receiving VRCZ were enrolled. There was no significant difference in the TAC C/D ratio for 7 days before and for the first and second 7-day periods after initiating LMV administration (median: 866 [IQR: 653-953], 842 [IQR: 636-1031], and 906 [IQR: 824-1210] [ng/mL]/[mg/kg], respectively). In contrast, the VRCZ C/D ratio and concentration for the first and second 7-day periods after LMV initiation were significantly lower than those before initiating LMV administration (mean 1.11 ± 0.07, 0.12 ± 0.08, and 0.22 ± 0.12 [μg/mL]/[mg/kg] and 0.7 ± 0.5, 0.8 ± 0.5, and 1.3 ± 0.7 μg/mL, respectively; n = 12). This can be explained by the increase in TAC concentration caused by CYP3A4 inhibition due to LMV and by the decrease in TAC concentration ascribed to the decrease in VRCZ concentration by CYP2C19 induction due to LMV. These results suggest that it is unnecessary to adjust the dose of TAC based on LMV initiation; however, it is necessary to adjust the dose of TAC based on conventional TAC concentration measurements.

## Introduction

Tacrolimus (TAC) has been widely used as an immunosuppressive drug for graft-versus-host disease (GVHD) prophylaxis after hematopoietic stem cell transplantation (HSCT) 1-6. GVHD remains the major cause of morbidity and mortality in HSCT recipients; therefore, the prevention of severe GVHD is crucial for its successful treatment 7. Because of the narrow therapeutic range and large intra- and inter-individual variabilities in the pharmacokinetics of TAC 8, adequate blood concentrations of TAC should be maintained to prevent drug-related toxicities and GVHD.

Although evidence regarding voriconazole (VRCZ) prophylaxis in patients receiving HSCT is poor 9, this drug is often used to prevent fungal infections. VRCZ is principally metabolized by cytochrome P450 2C19 (CYP2C19) and to a lesser extent by cytochrome P450 3A4 (CYP3A4) and cytochrome P450 2C9 (CYP2C9); it can also be an inhibitor of these enzymes 10-12. Because TAC is predominantly metabolized by CYP3A4 13, a significant interaction between VRCZ and TAC, that is, an increase in the whole blood concentration of TAC (TAC concentration) due to the inhibition of CYP3A4 by VRCZ, and the inhibition of TAC metabolism, dependent on the VRCZ concentration, have been reported 12,14-17.

Letermovir (LMV) is a new antiviral drug to prevent cytomegalovirus infection in HSCT recipients 18. It is a weak-to-moderate inhibitor of CYP3A4 and a weak-to-moderate inducer of CYP2C9 and CYP2C19 18. The interaction between LMV and TAC has been assessed in healthy participants, and LMV was found to increase TAC exposure 19,20. The interaction between LMV and VRCZ has also been assessed in healthy participants, and it was found that LMV decreases VRCZ exposure 21. Therefore, when LMV is administered to patients receiving TAC and VRCZ, a reciprocal effect of LMV on TAC concentration is supposed to occur due to the following: (1) an increase in the TAC concentration due to the inhibition of CYP3A4 by LMV and (2) a decrease in the TAC concentration ascribed to the decrease in VRCZ concentration due to the induction of CYP2C19 by LMV. If (1) is greater than (2), an increase in the TAC concentration is likely to occur, or if (2) is greater than (1), a decrease in the TAC concentration is likely to occur after the initiation of LMV administration. However, it is unknown which is greater, (1) or (2). Specifically, the effects of LMV on TAC in those who receive VRCZ are unknown. Therefore, in this study, we evaluated the effects of LMV on TAC pharmacokinetics by comparing the pharmacokinetic parameters before and after the initiation of LMV administration in HSCT recipients receiving VRCZ. Furthermore, we compared VRCZ pharmacokinetic parameters before and after the initiation of LMV administration to rationalize the participation of VRCZ in TAC pharmacokinetic changes.

## Patients and Methods

### Study design and patient population

This retrospective, observational, single-center study was conducted between May 2018 and April 2019 in Hyogo College of Medicine College Hospital in Japan by using medical chart reviews. Day 0 was defined as the day of the initiation of LMV. The pre-LMV period was defined as 7 days before the initiation of LMV (from day -6 to day 0). The post-LMV 1 period and post-LMV 2 period were defined as the first and second 7-day periods after the initiation of LMV (from day 1 to day 7 and from day 8 to day 14, respectively). The concentration/dose (C/D) ratio of TAC, plasma VRCZ concentration, and its C/D ratio during the pre-LMV period, post-LMV 1 period, and post-LMV 2 period were compared, respectively. The inclusion criteria were as follows: patients for whom LMV administration was initiated between May 2018 and April 2019, patients who received LMV once daily after breakfast (480 mg/day), patients who received TAC for the prophylaxis of GVHD, and patients who received VRCZ for the prophylaxis of a fungal infection. The following exclusion criteria were applied: patients for whom LMV was discontinued during the post-LMV 1 period or post-LMV 2 period; patients for whom VRCZ was initiated or discontinued during the pre-LMV period, post-LMV 1 period, or post-LMV 2 period; and patients for whom the VRCZ dose was changed during pre-LMV period, post-LMV 1 period, or post-LMV 2 period because TAC concentration is affected by the initiation or discontinuation of VRCZ.

### Evaluation of TAC concentration and C/D ratio

The TAC concentration was measured almost every day. Elecsys TAC assay kits were used to measure the TAC concentration using the Cobas e 801 analytical unit (Roche), according to the manufacturer's protocol. The daily dose of TAC was adjusted based on TAC concentration measurements, and the weight-adjusted dose (mg/kg per day) of TAC was calculated. To obtain the C/D ratio of TAC, the measured TAC concentration was divided by the corresponding dose administered 24 h prior to 6:00 a.m. on the day of the TAC concentration measurement per body weight on the day before the TAC concentration measurement. The C/D ratio of TAC during the pre-LMV period, post-LMV 1 period, and post-LMV 2 period was calculated as the mean value for each day during the three periods. The types and doses of proton pump inhibitors were surveyed because proton pump inhibitors increase the TAC concentration 22.

### Evaluation of plasma VRCZ concentration and C/D ratio

The plasma VRCZ concentration (VRCZ concentration) was measured almost weekly. VRCZ was administered orally or intravenously twice daily (10:00 a.m. and 20:00-22:00 p.m.) at a dose that was adjusted based on VRCZ concentration measurements. The VRCZ concentration was determined within 3 h before the administration of the next dose by liquid chromatography-tandem mass spectrometry, which was outsourced to SRL, Inc. (Tokyo, Japan). The VRCZ concentrations during the pre-LMV period, post-LMV 1 period, and post-LMV 2 period were defined as VRCZ concentrations during each of these respective periods. The period from VRCZ initiation to VRCZ concentration measurement was surveyed because it can affect the VRCZ concentration.

### Data collection

The following data at LMV initiation were collected from the electronic medical records: sex, age, height, body weight, disease requiring HSCT, source of stem cells, conditioning regimen, number of HLA mismatches, period from transplantation to LMV initiation, and clinical laboratory parameters (creatinine, total bilirubin, lactate dehydrogenase, aspartate aminotransferase, alanine aminotransferase, alkaline phosphatase, white blood cells, red blood cells, hemoglobin, hematocrit, and platelets).

### Statistical methods

The distribution of continuous variables was determined using the Kolmogorov-Smirnov normality test. Continuous data with a normal distribution and abnormal distribution are shown as mean ± standard deviation (SD) and median (interquartile range [IQR]) values, respectively. Categorical variables are shown as frequencies and percentages. Based on the results of the Kolmogorov-Smirnov normality test, repeated-measures analysis of variance (Bonferroni post hoc test) or Friedman tests were used to compare TAC C/D ratios, VRCZ C/D ratios, and VRCZ concentrations. The results with a probability value < 0.05 were considered significant. All analyses were conducted using SPSS statistics version 24.0 software (IBM, Tokyo, Japan).

### Ethics statement

The study protocol was approved by the Ethics Review Board of Hyogo College of Medicine (no. 3186). We obtained consent from all participants included in the study through an opt-out procedure.

## Results

### Patient characteristics

Seventeen patients met the inclusion criteria. Of these, three were excluded based on the exclusion criteria (for one, VRCZ was initiated during the pre-LMV period; for one, the VRCZ dose was changed during the post-LMV 1 period; for one, LMV was discontinued during the post-LMV 1 period), and 14 were enrolled in the study. Table 1 summarizes the clinical characteristics of individuals enrolled in this study and those for whom LMV administration was initiated. All patients received LMV orally. Table 2 summarizes the drugs administered concomitantly with LMV and VRCZ at LMV initiation.

### TAC C/D ratio

There were no significant differences in the C/D ratios of TAC during the pre-LMV period, post-LMV 1 period, and post-LMV 2 period (Table 3). All patients received proton pump inhibitors orally. The types and doses of proton pump inhibitors were the same during the pre-LMV period, post-LMV 1 period, and post-LMV 2 period.

### VRCZ C/D ratio and concentration

Of the 14 patients enrolled in the study, the VRCZ concentration was measured in 12 patients during the pre-LMV period, post-LMV 1 period, and post-LMV 2 period (all patients received oral VRCZ). The mean C/D ratio of VRCZ during the post-LMV 1 period and post-LMV 2 period was significantly lower than that during the pre-LMV period. The mean VRCZ concentration during the post-LMV 1 period and post-LMV 2 period was significantly lower than that during the pre-LMV period (Table 3). In two, six, three, and one patient(s), the VRCZ concentration during the pre-LMV period was measured on day -4, -3, -1, and 0, respectively. In two, five, four, and one patient(s), the VRCZ concentration during the post-LMV 1 period was measured on day 3, 4, 6, and 7, respectively. In two, six, three, and one patient(s), the VRCZ concentration during the post-LMV 2 period was measured on day 10, 11, 13, and 14, respectively. The period from VRCZ initiation to VRCZ measurement during the pre-LMV period was more than 14 days for all patients.

## Discussion

To the best of our knowledge, this is the first study to assess the effect of LMV on TAC in HSCT recipients receiving VRCZ. The results demonstrated that there was no significant difference in the C/D ratios of TAC before and after LMV initiation. This can be explained by the increase in the TAC concentration by CYP3A4 inhibition due to LMV and by the decrease in TAC concentration by CYP2C19 induction due to LMV.

It is important to evaluate the interaction at the time when the enzyme is sufficiently inhibited or induced. It is generally assumed that enzyme inducers accelerate enzyme synthesis in a concentration-dependent manner. Since enzyme synthesis is assumed to obey zero-order kinetics, the effect of the inducer on enzyme synthesis starts immediately. Therefore, the gradual increase in CYP activity over several days of exposure to the inducer is attributed simply to the slow degradation rate of these enzymes 23. Stable trough levels of LMV were reached on day 4 after LMV initiation 24. However, several studies have indicated that it takes at least 24 to 72 h for the CYP activity of the enzyme inducer to reach a maximum level 25,26. It is unclear when CYP2C19 induction due to LMV reaches a maximum level. However, in a pharmacokinetic trace of LMV co-administered with VRCZ, the pharmacokinetics were assessed on day 8 after the 7-day administration of LMV 21. Therefore, in the present study, the interaction was evaluated during the pre-LMV period, post-LMV 1 period, and post-LMV 2 period (each for 7 days).

In a previous study, the co-administration of LMV at clinical doses with TAC resulted in a 2.4-fold increase in the area under the plasma TAC concentration-time curve and a 1.6-fold increase in the maximum plasma TAC concentration in 14 healthy female participants 20. This was because TAC is predominantly metabolized by CYP3A4 13 and LMV is a moderate inhibitor of CYP3A4 18. In contrast, the effect of LMV on VRCZ was examined in 14 healthy subjects 21. In that study, the area under the curve and peak concentration geometric mean ratios for VRCZ+LMV/VRCZ alone were 0.56 and 0.61, respectively. This might be because VRCZ is eliminated largely via hepatic metabolism by CYP2C19, with a minor contribution from CYP3A4 and CYP2C9 10,11, and LMV induces weak-to-moderate CYP2C19 and CYP2C9 activities 18. Moreover, the inhibition of TAC metabolism is positively dependent on the VRCZ concentration due to the inhibition of CYP3A4 by VRCZ 15-17. Guo et al. reported three cases in which TAC and LMV were administered 27. In their study, two cases without VRCZ administration showed a significant increase in the TAC concentration; however, the TAC concentration did not fluctuate greatly in one case with VRCZ administration. The findings in these cases seemed to support our results.

Regarding VRCZ, an analysis of the exposure-response relationship indicated that the optimal target plasma trough concentrations ranged between 1.5 and 4.5 μg/mL 28 and therapeutic drug monitoring is recommended to maximize therapeutic success 29. However, the present study showed a considerable decrease in VRCZ concentrations after LMV initiation, and the concentration decreased to levels lower than optimal target plasma trough concentrations. Thus, frequent measurements of VRCZ concentrations might be necessary to maintain the optimal target plasma trough concentration range when LMV is initiated in patients receiving VRCZ.

Our study has several limitations. First, the present study had a retrospective observation design without a control group and not as an intervention trial. Thus, the magnitude of interactions was not considered. Second, in this study, polymorphisms in genes encoding metabolic enzymes (CYP3A4, CYP3A5, CYP2C9, and CYP2C19) were not assessed. In one participant, the C/D ratios of TAC were >2000 (ng/mL)/(mg/kg). This might be affected by gene polymorphisms. Further studies are required to address this.

## Conclusion

The present study demonstrated that the C/D ratio of TAC does not significantly change and that the VRCZ concentration significantly decreases after the initiation of LMV administration.

## Figures and Tables

**Table 1:** Patient and transplantation characteristics

**Patients, *n***	14
**Men, *n* (%)**	11 (79)
**Age, years**	44 ± 11
**Height, cm**	172 (167, 176)
**Body weight, kg**	62.9 ± 8.6
**Disease**	
Acute myeloid leukemia, *n* (%)	5 (36)
Acute lymphocytic leukemia, *n* (%)	4 (29)
Myelodysplastic syndromes, *n* (%)	2 (14)
Lymphoblastic lymphoma, *n* (%)	2 (14)
Diffuse large B-cell lymphoma, *n* (%)	1 (7)
**Source of stem cells**	
Peripheral blood, *n* (%)	13 (93)
Bone marrow, *n* (%)	1 (7)
**Conditioning regimen**	
Myeloablative, *n* (%)	1 (7)
Reduced intensity, *n* (%)	13 (93)
**Number of HLA mismatches**	
1, *n* (%)	1 (7)
2, *n* (%)	0 (0)
≥ 3, n (%)	13 (93)
**Period from transplantation to LMV initiation, days**	3 (3, 4)
**Creatinine, mg/dL**	0.58 (0.41, 0.86)
**Total bilirubin, mg/dL**	0.4 (0.3, 0.9)
**Lactate dehydrogenase, IU/L**	235 (169, 292)
**Aspartate aminotransferase, IU/L**	19 ± 9
**Alanine aminotransferase, median, IU/L**	21 (14, 32)
**Alkaline phosphatase, IU/L**	255 ± 65
**White blood cell, /μL**	165 (50, 300)
**Red blood cell, ×10^4^/μL**	289 (270, 299)
**Hemoglobin, g/dL**	8.8 ± 0.8
**Hematocrit, %**	25.2 ± 2.4
**Platelet, ×10^4^/μL**	3.5 (2.7, 4.8)
**Route of voriconazole administration**	
Oral administration, *n* (%)	13 (93)
Drip infusion, *n* (%)	1 (7)

Data are expressed as Data are expressed as mean ± SD for normally distributed continuous variables, median (25, 75% interquartile range) for abnormal distributed continuous variables or number (percentage).

**Table 2:** Drugs administered concomitantly with LMV and VRCZ at LMV initiation

**Antiviral agent**	
Acyclovir, *n* (%)	14 (100)
**Antimicrobial agent**	
Moxifloxacin hydrochloride, *n* (%)	13 (93)
Meropenem, *n* (%)	12 (86)
Tazobactam/piperacillin, *n* (%)	2 (14)
Linezolid, *n* (%)	6 (43)
**Antifungal agent**	
Caspofungin, *n* (%)	8 (57)
**Proton pump inhibitor**	
Lansoprazole, *n* (%)	11 (79)
Esomeprazole, *n* (%)	2 (14)
**Corticosteroid**	
Methylprednisolone, *n* (%)	11 (79)
Prednisolone, *n* (%)	2 (14)
**Other**	
Ursodeoxycholic acid, *n* (%)	14 (100)
Lenograstim, *n* (%)	10 (71)
Danaparoid sodium, *n* (%)	9 (64)
Amlodipine, *n* (%)	3 (21)
Brotizolam, *n* (%)	2 (14)
Zolpidem, *n* (%)	2 (14)
Furosemide, *n* (%)	2 (14)

Data do not include infusions. Each one patient received atovaquone, pregabalin, alendronate, polaprezinc, L-carbocisteine, fexofenadine, magnesium oxide, febuxostat, sitagliptin, rabeprazole, levofloxacin, *Lactobacillus* preparation, daptomycin, aztreonam, metoclopramide, defibrotide, carperitide, teicoplanin, panthenol, and liposomal amphotericin B.

**Table 3:** TAC C/D ratio, VRCZ C/D ratio, and VRCZ concentration before and after LMV initiation

	Pre-LMV period	Post-LMV 1 period	Post-LMV 2 period	p value
**TAC C/D ratio, (ng/mL)/(mg/kg)**	866 (653, 953)	842 (636, 1031)	906 (824, 1210)	0.931
**VRCZ C/D ratio, (µg/mL)/(mg/kg)**	0.22 ± 0.12	0.11 ± 0.07	0.12 ± 0.08	0.005
p value (vs pre-LMV period)		0.029	0.007	
p value (vs post-LMV 1 period)			1.000	
**VRCZ concentration, µg/mL**	1.3 ± 0.7	0.7 ± 0.5	0.8 ± 0.5	0.003
p value (vs pre-LMV period)		0.023	0.006	
p value (vs post-LMV 1 period)			1.000	

LMV: letermovir; VRCZ: voriconazole; C/D: concentration/dose

## Abbreviations

C/D: concentration/dose
CYP: cytochrome P450
GVHD: graft-versus-host disease
HSCT: hematopoietic stem cell transplantation
IQR: interquartile range
LMV: letermovir
SD: standard deviation
TAC: tacrolimus
VRCZ: voriconazole
